# Supplementary material for: Causal Relationship between Aging and Anorexia Nervosa: A White-Matter-Microstructure-Mediated Mendelian Randomization Analysis
Source: Biomedicines. 2024 Aug 16;12(8):1874. doi: 10.3390/biomedicines12081874 (PMC11351342; doi:10.3390/biomedicines12081874)
Supplement: Supplementary file 1 [file biomedicines-12-01874-s001.zip › Supplementary Figure/Figure S7/Single SNP plot of MR analysis for ubm-b-1952 to AN.pdf]

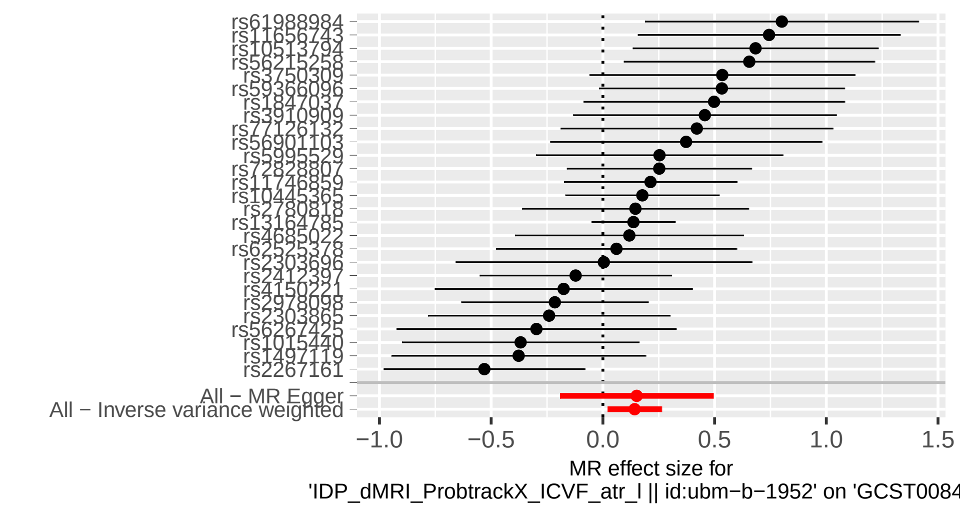
rs61988984
rs11656743
rs10513794
rs56215258
rs3750309
rs59366096
rs1847037
rs3910909
rs77126132
rs56901103
rs5995529
rs72828807
rs11746859
rs10445365
rs2780818
rs13164785
rs4685022
rs62525378
rs2303696
rs2412397
rs4150221
rs2978098
rs2303865
rs56267425
rs1015440
rs1497119
rs2267161
All − MR Egger
All − Inverse variance weighted
−1.0
−0.5
0.0
0.5
1.0
1.5
MR effect size for
'IDP_dMRI_ProbtrackX_ICVF_atr_l || id:ubm−b−1952' on 'GCST0084
